# Dissimilar Roles of the Four Conserved Acidic Residues in the Thermal Stability of Poly(A)-Specific Ribonuclease

**DOI:** 10.3390/ijms12052901

**Published:** 2011-05-03

**Authors:** Guang-Jun He, Wei-Feng Liu, Yong-Bin Yan

**Affiliations:** State Key Laboratory of Biomembrane and Membrane Biotechnology, School of Life Sciences, Tsinghua University, Beijing 100084, China; E-Mails: he-gj06@mails.tsinghua.edu.cn (G.-J.H.); liuwf@mail.tsinghua.edu.cn (W.-F.L.)

**Keywords:** aggregation kinetics, magnesium ions, poly(A)-specific ribonuclease (PARN), structural stability, thermal aggregation, two-metal-ion catalysis

## Abstract

Divalent metal ions are essential for the efficient catalysis and structural stability of many nucleotidyl-transfer enzymes. Poly(A)-specific ribonuclease (PARN) belongs to the DEDD superfamily of 3′-exonucleases, and the active site of PARN contains four conserved acidic amino acid residues that coordinate two Mg^2+^ ions. In this research, we studied the roles of these four acidic residues in PARN thermal stability by mutational analysis. It was found that Mg^2+^ significantly decreased the rate but increased the aggregate size of the 54 kDa wild-type PARN in a concentration-dependent manner. All of the four mutants decreased PARN thermal aggregation, while the aggregation kinetics of the mutants exhibited dissimilar Mg^2+^-dependent behavior. A comparison of the kinetic parameters indicated that Asp28 was the most crucial one to the binding of the two Mg^2+^ ions, while metal B might be more important in PARN structural stability. The spectroscopic and aggregation results also suggested that the alterations in the active site structure by metal binding or mutations might lead to a global conformational change of the PARN molecule.

## 1. Introduction

Divalent metal ions are essential for the efficient catalysis of many nucleotidyl-transfer enzymes including polymerases, nucleases and phosphotases. Most of these metalloenzymes can be classified into two groups: Some utilize the two-metal-ion catalytic mechanism, while the others use only one metal ion for catalysis. The two-metal-ion mechanism was first proposed for the 3′–5′ exonuclease of the Klenow fragment of *Escherichia coli* DNA polymerase I [1], and has been characterized in a number of phosphoryl transferases [2]. In the catalysis of these enzymes, one metal ion (denoted as metal ion A) activates a water molecule and orients it to attack the scissile phosphate, while metal ion B helps to stabilize the reaction intermediate and facilitates leaving the 3′-OH group [1,3]. In the one-metal-ion catalysis, a single metal ion binds to the enzyme. It was proposed very recently that this metal ion is catalytically equivalent to metal ion B of two-metal-ion catalysis, and the function of metal A was replaced by an amino acid residue acting as the general base [4]. Compared to the mononuclear enzyme, the physical and chemical features of the binuclear center endow the enzyme using a two-metal-ion mechanism with higher metal-ion preference, catalytic specificity and versatility [2,4,5]. Unlike the low metal-ion preference of mononuclear enzymes, Mg^2+^ is usually the most favorable one for binuclear enzymes [2,6].

Although a growing number of enzymes are found to follow the two-metal-ion catalysis, the detailed mechanism is far from fully understood [2,6,7]. Particularly, the individual contributions of the two metal ions remain elusive. According to the current model, metal ion A plays a crucial role in catalysis, while metal ion B binds much more weakly than metal ion A and may play a regulatory role in some binuclear enzymes [6,7]. However, the residue coordinated metal ion B is found to be more sensitive to mutations in *Bacillus halodurans* RNase H [8]. Moreover, the numbers and locations of the two metal ions differ significantly in the crystal structures of some enzyme families [7], which may be due to the relative weaker binding constants, alternation of active site structure or metal movement during catalysis. [2,7]. Nevertheless, it is clear that the metal ion cofactors are crucial not only to the efficient catalysis of enzymes, but also to their structural stability (for example, [9–13]). An interesting and unresolved question is whether the two functionally non-equivalent metal ions have different contributions to enzyme stability.

**Figure 1.**
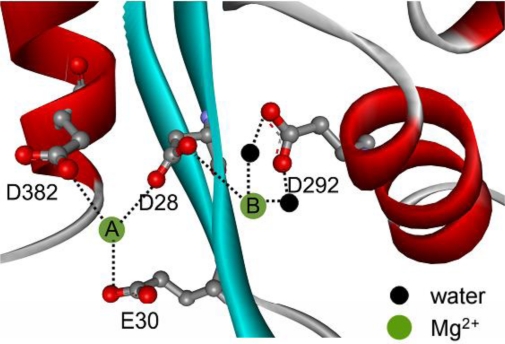
Structure of the active site of PARN (adapted from reference [18] and PDB ID 2A1S). The four conserved acidic residues are highlighted by ball-and-stick model. The Mg^2+^ binding sites are predicted on the basis of previous studies [16,18]. The dashed lines indicate the possible interaction network that stabilizes the two Mg^2+^ ions. For clarity, the substrate molecule was not shown.

Poly(A)-specific ribonuclease (PARN, EC 3.1.13.4) is involved in the mRNA decay regulation by specifically catalyzing the shortening of the 3′-end poly(A) tail [14,15]. The full length PARN contains three well-defined domains: The catalytic domain, the R3H domain and the RNA-recognition motif (RRM). The catalytic nuclease domain belongs to the DEDD superfamily of 3′-exonucleases, and shares a similar conserved core structure to the other members in this superfamily [16]. The active site of PARN contains four conserved acidic amino acid residues, D28, E30, D292 and D382, which are essential for the catalytic activity of PARN [17,18]. These acidic residues form a negative charge cave that can bind with two Mg^2+^ ions (Figure 1). It has been proposed that PARN may utilize the general two-metal ion mechanism for catalysis based on biochemical [18] and structural analysis [16] although no metal ion is present in the crystal structures of ligand-free and substrate-bound PARN [16]. The cofactor Mg^2+^ is also important to PARN stability against inactivation induced by heat treatment, but promotes thermal aggregation at high temperatures [12]. Because mutation of any of the four conserved acidic residues in the active site will inactivate PARN, we evaluated the roles of the two Mg^2+^ ions in PARN stability by thermal aggregation of the wild type (WT) and mutated enzymes. The data indicated that all of the four mutants decreased PARN thermal aggregation. The aggregation kinetics of the mutants exhibited dissimilar Mg^2+^ dependence, suggesting that these four conserved acidic residues played differential roles in Mg^2+^ coordination and protein stability.

## 2. Results and Discussion

### 2.1. Effects of Mutations on PARN Activity and Structure

All of the four recombinant mutated proteins were eluted at the same volume as the WT in the size-exclusion chromatography (SEC) profile (data not shown), indicating that the mutations of the four conserved acidic residues, D28, E30, D292 and D382, did not affect the oligomeric state of PARN. To investigate the effect of mutation on PARN function, the deadenylase activity was assayed by the methylene blue method [19]. Consistent with the previous observations [18], all four of the mutants were found to be completely inactive, suggesting that these four conserved acidic residues were essential for the two-metal-ion catalysis of PARN.

**Figure 2.**
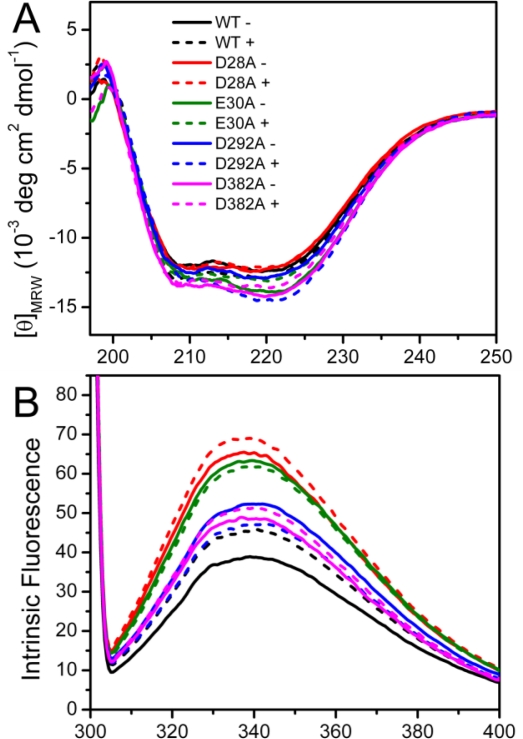

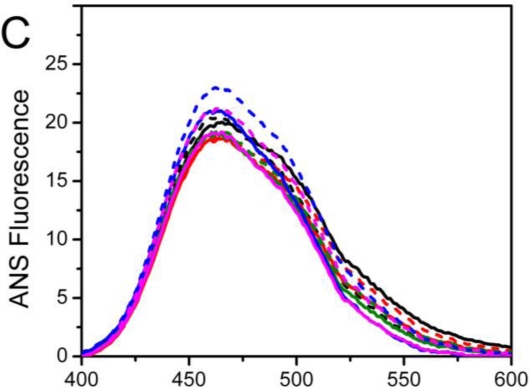
Effects of mutations on PARN secondary and tertiary structures monitored by far-UV CD (**A**), intrinsic Trp fluorescence (**B**) and ANS fluorescence (**C**). The proteins were dissolved in buffer T containing 20 mM Tris-HCl (pH 8.0), 100 mM KCl, 0.5 mM DTT, 0.2 mM EDTA and 20% (v/v) glycerol in the presence or absence of 3 mM MgCl_2_. The final protein concentration was 0.2 mg/mL. The excitation wavelength of the intrinsic fluorescence was 295 nm, while that of the ANS fluorescence was 380 nm. The resultant spectra were obtained by the subtraction of the spectra of the buffer. All spectroscopic experiments were carried out at 25 °C.

Spectroscopic experiments were carried out to assess the effect of mutation on PARN structure. The circular dichroism (CD) data shown in Figure 2A indicated that the effects of the mutations on the secondary structure contents of PARN were minor. The four mutations affect PARN secondary structures dissimilarly: No significant changes were observed for the D28A and D292A mutations, while there was an ∼10% increase in the absolute value of ellipticity at 222 nm ([*θ*_222_]) for the E30A and D382A mutations. With the addition of 3 mM Mg^2+^, a minor increase of [*θ*_222_] was observed for the WT protein. As for the mutants, the [*θ*_222_] value changed little for D28A; there was a slight decrease for D382A and a significant decrease to the value close to that of the WT protein for E30A. However an ∼15% increase was found for D292A. These results indicated that the mutations either slightly increased or did not affect the percentages of the regular secondary structure contents of PARN. The coordination of Mg^2+^ could rescue the disturbance in secondary structures induced by most mutations except D292A.

Intrinsic (Figure 2B) and 1-anilinonaphtalene-8-sulfonate (ANS) fluorescence (Figure 2C) were used to probe the effects of mutations on the PARN tertiary structure. When excited at 295 nm, the intrinsic fluorescence mainly reflects the microenvironmental status of Trp fluorophores. Consistent with previous observations [20], the wavelength of the maximum Trp fluorescence was around 340 nm for both of the samples with and without Mg^2+^. The mutations did not alter the maximum Trp fluorescence wavelength or shape of the spectra, suggesting that the mutations had no significant effect on the solvent accessibility of PARN Trp residues. Interestingly, the intrinsic fluorescence intensity was significantly increased due to the mutations. Because many factors may affect the fluorescence yield, it is difficult to determine accurately the reason for the intensity changes of intrinsic fluorescence. However, it is clear that the cofactor binding or mutation induced changes in the microenvironments around Trp residues.

Among the four mutants, the D28A and E30A mutations resulted in an ∼2-fold increase in Trp fluorescence, while the D292A and D382A mutations led to an ∼30% increase. With the addition of Mg^2+^, an increase in the Trp fluorescence intensity was observed for the WT protein (17%), D28A (6%) and D382A (5%); there was a slight quenching for E30A (3%) and D292A (10%). Meanwhile, the ANS fluorescence indicated that most of the mutants possessed similar ANS-binding ability to the WT PARN under both Mg^2+^-free and Mg^2+^-bound conditions, except that the Mg^2+^-bound D292A had a relative larger hydrophobic exposure.

Taken together, the minor difference in the CD signal, maximum Trp fluorescence wavelength and ANS fluorescence suggested that the mutations slightly modified PARN secondary and tertiary structures. The four mutations had dissimilar effects on PARN structure. Among them, D28A and E30A had similar secondary structures and hydrophobic exposure to the WT but great increase in Trp fluorescence intensity, while the D292A and D382A mutations resulted in the formation of more regular secondary structures, moderate increase in Trp fluorescence intensity, and different behavior upon Mg^2+^ coordination compared with the WT protein.

### 2.2. Effect of Mg^2+^ on PARN Thermal Aggregation

**Figure 3.**
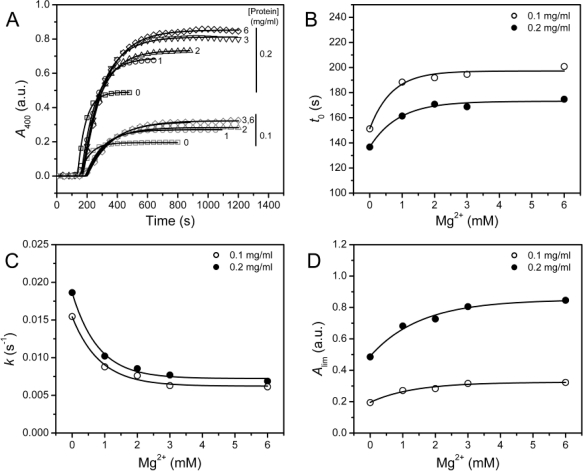
(**A**) Representative time-course aggregation during PARN heated at 55 °C in the presence of various amounts of Mg^2+^. The aggregation was monitored by the absorbance at 400 nm on an Ultraspec 4300 pro UV/Visible spectrophotometer. The final protein concentration was 0.1 or 0.2 mg/mL. The raw data, which are presented as open symbols, were recorded every 2 s. The fitted curves are drawn as solid lines. The concentrations of Mg^2+^ (0–6 mM) and PARN are labeled. (**B**–**D**) Mg^2+^ dependence of the aggregation kinetic parameters. The parameters were obtained by fitting the raw data with Equation (1). The errors of the fitted parameters were within 5%.

In a previous study, we found that 3 mM Mg^2+^ can protect PARN against thermal inactivation below 60 °C, but it accelerates protein thermal aggregation at 61 °C [12]. A similar result was observed when PARN was heated at 55 °C (Figure 3A), where PARN thermal aggregation revealed Mg^2+^- and protein-concentration dependent manner. The aggregation kinetics was analyzed by considering the protein aggregation process as an *n*-th order reaction, and the following equation could be used for the fitting of the data [21]

(1) $$A\;=\;A_{\text{lim}}\;(1\;-\;\text{exp}(-k_{n}{\left(t\;-\;t_{0}\right)}^{n})$$

where *t* is the time of incubation at a given temperature, *A*_lim_ is the *A*_400_ value at the infinite time and *k*_n_ is the rate constant of the *n*-th order reaction. Under all conditions, the aggregation was found to be dominated by a first-order kinetics with *n* = 1.17 ± 0.08. To facilitate the comparison of the parameters, all the data were fitted by assuming *n* = 1. The results shown in Figure 3 indicated that all the three parameters (*t*_0_, *k* and *A*_lim_) were significantly affected by the addition of Mg^2+^ (*F* test, *P* < 0.0001). Unexpectedly, the three parameters revealed inconsistent results with the increase of [Mg^2+^]: The increase in *t*_0_ and the decrease in *k* implied that Mg^2+^ could inhibit the rate of PARN aggregation, whereas the increase in *A*_lim_ suggested that Mg^2+^ increased the amounts or size of aggregates.

**Figure 4.**
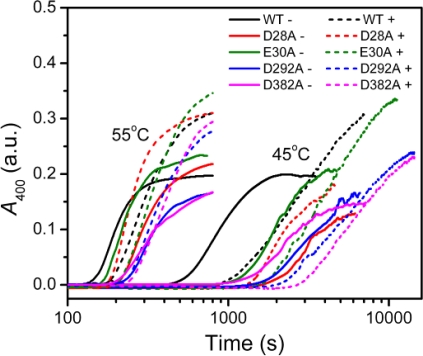
Effects of mutations on PARN thermal aggregation at 45 °C (A) and 55 °C (B) in the presence or absence of 3 mM Mg^2+^. The experimental details were the same as those described in Figure 3. For clarity, the fitted curves were not shown.

Turbidity, which was reflected by the absorbance at 400 nm in this research, is an indicator of the size of the aggregates [22]. Since most proteins were found to exist in the aggregates after heat treatment, which were evidenced by the lack of soluble fractions when assayed by either spectroscopic methods or SDS-PAGE (data not shown), the increase of *A*_lim_ suggested that the protein might aggregate into larger forms in the presence of Mg^2+^. Because the inactivation of PARN was much faster than its aggregation, the observations herein might explain the different effects of Mg^2+^ on PARN inactivation and aggregation observed previously [12]. The results shown in Figure 3 also suggested that great caution should be taken when using *A*_lim_ alone to evaluate protein aggregation. To confirm the results in Figure 3, the aggregation of PARN was also performed at 45 °C, and similar results were obtained except that aggregation process was significantly slowed down by the decrease of temperature (data not shown, see also Figure 4). Thus both the variations in the protein concentration and incubating temperature did not alter the Mg^2+^-dependence of PARN thermal aggregation.

**Figure 5.**
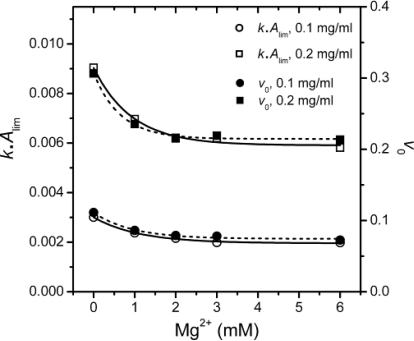
Mg^2+^ dependence of PARN thermal aggregation at 55 °C evaluated by *kA*_lim_ and the initial velocities of aggregation (*v*_0_). The fitted curves were obtained from Equation (2).

The inconsistency between *k* and *A*_lim_ shown in Figure 3 was also observed in the study of chaperone functions [21]. It has been suggested that the value *kA*_lim_, which reflects the initial velocity of aggregation, is a better indicator to measure the effects of cosolvents on first-order protein aggregation [21]. The Mg^2+^-dependence of the *kA*_lim_ values as well as the initial velocities of aggregation (*v*_0_) were calculated and presented in Figure 5.

It is clear that the *kA*_lim_ and *v*_0_ values coincided with each other, and the results also indicated that Mg^2+^ could decrease PARN thermal aggregation in a concentration dependent manner. The Mg^2+^ dependence for the *kA*_lim_ data could be regarded as ligand-binding induced protein aggregation. Thus, the data in Figure 5 could be fitted by the following equation

(2) $$\Delta Y\;=\;\Delta Y_{\text{max}}[{\text{Mg}}^{2+}]/(appK_{\text{D}}\;+\;[{\text{Mg}}^{2+}])$$

where Δ*Y* is the relative change of the *kA*_lim_ value, and *appK*_D_ is the apparent dissociation constants of the aggregate·ligand complex. It is worth noting that the *appK*_D_ value only reflects the Mg^2+^-dependence for PARN aggregation, but is not directly related to the binding of Mg^2+^ to the aggregates. The *appK*_D_ values were 0.9 ± 0.2 and 0.7 ± 0.1 mM for 0.1 and 0.2 mg/mL PARN aggregation, respectively. Similar values could also be obtained from the data shown in Figures 3B–3D. The small *appK*_D_ values of the WT PARN indicated the aggregation of the WT protein had a strong dependence on the existence of Mg^2+^. This value is close to the optimal Mg^2+^ concentration required for the catalysis of the 54 kDa PARN (∼1 mM) [23], suggesting that the effects of Mg^2+^ on PARN aggregation was related to Mg^2+^-coordination in the active site.

### 2.3. Effect of Mutations on PARN Thermal Aggregation

**Figure 6.**
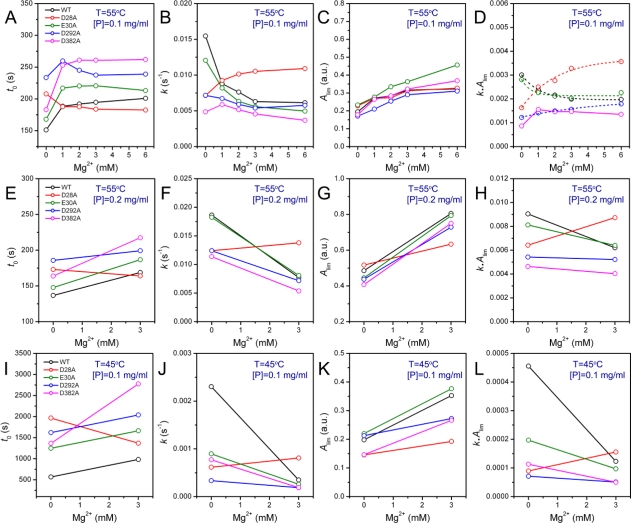
Mg^2+^ dependence of the kinetic parameters of the WT and mutated PARN thermal aggregation at 55 °C (**A**–**H**) or 45 °C (**I**–**L**). The protein concentrations were 0.1 (**A**–**D** and **I**–**L**) or 0.2 mg/mL (**E**–**H**).

The thermal aggregation of PARN was [Mg^2+^] dependent, and reached an equilibrium at [Mg^2+^] > 3 mM. This suggested that the coordination of Mg^2+^ to the active site might be correlated to the effects of Mg^2+^ on PARN thermal aggregation. To elucidate this proposal, mutational analysis was carried out with the four conserved acidic residues that directly participated in Mg^2+^-binding (Figure 1). The results indicated that in the absence of Mg^2+^, all four mutations significantly decreased PARN aggregation at both 45 °C and 55 °C (Figures 4 and 6). The aggregation of all mutants exhibited similar temperature-dependence to the WT protein. That is, with the decrease of temperature, the aggregation had a significantly longer *t*_0_, a smaller *k* and an almost unchanged *A*_lim_. When protein concentration was increased from 0.1 to 0.2 mg/mL, the changes in *t*_0_ and *k* were minor, while an about 2-fold increase in *A*_lim_ was observed for all proteins.

As presented in Figure 6, the addition of Mg^2+^ increased the *A*_lim_ values of all proteins. However, the Mg^2+^ dependence of the other kinetic parameters was dramatically different. Among the four mutants, the E30A mutation resulted in the least changes in the aggregation of PARN under all experimental conditions, and its Mg^2+^-dependent property was very similar to the WT protein except that E30A aggregated slower. The most striking observation is that D28A revealed reverse Mg^2+^ dependence when compared to that of the WT protein. In the absence of Mg^2+^, the E28A mutation decreased PARN aggregation, which is similar to the other three mutants. However, in contrast to the aggregate-inhibition effects of Mg^2+^ on the WT and the other three mutants, the addition of Mg^2+^ significantly increased PARN aggregation by decreasing *t*_0_ and increasing *k*, *A*_lim_ and *kA*_lim_. In the presence of 3 mM Mg^2+^, the aggregation of D28A was more serious than the WT protein. In most circumstances, D292A and D382A were the two most effective mutations in inhibiting PARN thermal aggregation. The performance of D292A and D382A was between that of D28A and E30A. The Mg^2+^ dependence of D382A was close to that of E30A for most cases, while D292A was somewhat more like D28A. It is worth noting that the impact of Mg^2+^ on D292A thermal aggregation was weak and was not as obvious as the other three mutants.

### 2.4. Discussion

The structural stability of multimeric/multi-domain proteins depends not only on the properties of individual domains but also on the domain interactions [24,25], and thus protein stability analysis can also provide insights into protein structure and regulation. The full-length PARN is a dimeric enzyme containing three well-defined domains: The nuclease domain, the R3H domain and the RRM [16]. In addition to the well-folded domains, the C-terminal domain of PARN has recently been proposed to be intrinsically disordered [26]. The 54 kDa isoform is the N-terminal proteolytic product of the full-length PARN [23] with the removal of the C-terminal domain and half of the RRM [20]. The 54 kDa PARN contains two Trp residues: W219 at the R3H domain and W456 at the truncated RRM domain [20]. Although no Trp residue is located at the catalytic nuclease domain, the significant changes in the Trp fluorescence induced by Mg^2+^ coordination (Figure 2) strongly suggested that the conformation of the other domains was affected by structural changes of the active site. That is, the domain-interaction interface might be close to the active site. This opinion was confirmed by mutational analysis, which indicated that all four mutations at the active site resulted in a dramatic increase in the fluorescence intensity. Indeed, very recently, structural analysis indicated that the RRM interacts with the catalytic domain [27]. Our results further suggested that Mg^2+^ coordinated in the active site not only played an essential role in the two-metal catalysis of PARN, but also induced a large scale conformational change. Although the maximum Trp fluorescence wavelength was not affected by Mg^2+^-coordination or mutations, the increase of Trp fluorescence intensity implied that the cofactor or mutation decreased the flexibility around the Trp residues. Meanwhile, a slight increase in the ellipticity was also observed for Mg^2+^-coordination and most mutations except D28A. Thus these spectroscopic results suggested that the Mg^2+^ coordination or mutations might modulate the repulsion of the negative charges of the four acidic residues in the active site, which further induced large conformational changes of the PARN molecule to a more compact one. Although this conclusion was obtained by studies of the 54 kDa PARN isoform, it could also be true for the 74 kDa full length PARN. In the DEDD nucleases, a fifth residue, which is either His or Tyr, also contributes to the enzymatic activity. PARN belongs to the DEDDh subfamily, and His337 is the fifth residue. However, His337 does not contribute to the coordination of the metal ions, and may not involve in the structural changes induced by the addition of Mg^2+^. Moreover, both the addition of Mg^2+^ and mutations of the four acidic residues significantly decreased PARN thermal aggregation (Figure 6), although the active site is buried in the catalytic domain and seems not to participate in PARN aggregation [28]. Considering that PARN is an allosteric enzyme [29], it is important to find that the Mg^2+^ coordination could induce conformational changes in the other domains of PARN, which might contribute to the allosteric regulation of PARN. This conclusion is quite consistent with the previous observations by Balatsos *et al*., which showed that the coordination of Mg^2+^ could release the non-competitive nucleotide inhibitors of PARN [30]. Our findings is also consistent with that previously observed by Dupureur and Dominguez, which showed that the mutations in the active site of *Pvu*II endonuclease resulted in significant changes in the conformation and stability of the enzyme [13]. Particularly, they found that three of the mutations led to a 2–5 kcal/mol stabilization of *Pvu*II endonuclease, implying that the mutants might have a more compact structure.

In most cases, the binding of the divalent metal ion cofactor stabilizes the protein against stresses. Previous research has indicated that Mg^2+^ can retard PARN thermal inactivation, but promote PARN aggregation evidenced by the increase of turbidity [12]. We found that Mg^2+^ had dissimilar effects on the kinetic parameters of PARN thermal aggregation. It is worth noting that under the same protein concentration and incubating conditions, the *A*_lim_ value reflects the size of the aggregates as proposed previously [22]. Thus Mg^2+^ decreased the rate of the aggregation, but increased the size of aggregates. This unique effect might be caused by the interference of PARN aggregation pathway by Mg^2+^. The little change in secondary and tertiary structures [12] and the first-order kinetics (Figure 3) suggested that PARN thermal aggregation was dominated by an unfolding-limited aggregation mechanism (native ↔ unfolded/intermediate → aggregate) [31]. The fact that Mg^2+^ elongated *t*_0_ of the WT protein indicated that the cofactor binding stabilized the active site or native structure, and delayed the unfolding process of PARN. Meanwhile, a decrease in *k* suggested that the Mg^2+^ might also alter the properties of the intermediate state. The Mg^2+^-modified intermediates were prone to form larger aggregates when compared to the apo-protein. The underlying molecular mechanism remains unclear. A possible explanation is that Mg^2+^ modulated the negative charge repulsion and facilitated the formation of larger aggregates. Actually, electrostatic interactions have been proposed to contribute to protein aggregation for many proteins [32–36], and PARN aggregation could also be modulated by electrostatic interactions during chemical denaturant-induced denaturation [26].

The aggregation of the four mutants showed quite dissimilar Mg^2+^-dependent properties, which might be due to their differential roles in Mg^2+^ coordination. It has been proposed that in PARN catalysis, metal ion A is coordinated by D28, E30 and D382, while metal ion B binds with D28 and D292 (Figure 1) [17,18]. Obviously, D28 is the most important one in Mg^2+^ coordination of PARN, and it has been observed that among the four acidic residues, D28 is crucial to Fe^2+^ coordination when substituted by Ala [17]. Consistent with this observation, we found that the aggregation of D28A showed a reverse Mg^2+^ dependent property when compared to the WT PARN, and this property was not observed or not obvious in the other mutants (Figures 6). The reason why the D28A mutation had the unique property of reversing the action of Mg^2+^ is unclear. A shorter *t*_0_ and a larger *k* implied that the addition of Mg^2+^ altered both the unfolding and the aggregation stage in the unfolding-limited aggregation of D28A. A possible reason is that the mutation disrupted the correct positioning of both metal A and metal B, and led a change in the active site structure. The misfolded active site, as proposed above, resulted in a global change in PARN conformation and facilitated protein aggregation.

If the two metal ions contributed differently to PARN thermal stability, the behavior of D292A should be different from those of and D382A or E30A. In most cases, the Mg^2+^-dependence of D292A aggregation was not as obvious as the other mutants. Meanwhile, the E30A mutant behaved just similar to the WT protein, while the property of D382A was between those of D292A and E30A (Figure 6). Considering that D292 participates the binding of metal B via two water molecules [17,18], the significant changes in Mg^2+^ dependence by mutation suggested that weakening the binding of metal B resulted in a more serious disruption of the active site structure. Thus the coordination of metal B (Figure 1) might be more important to the structural stability of PARN. This might also be the reason why the effect of the D28A mutation was not the sum of that of D292A and D382A/E30A since D28 coordinates metal B directly by its side chains. It is worth noting that metal B in enzymes utilizing two-metal-ion catalysis is catalytically equivalent to the single metal ion in enzymes with one-metal-ion catalysis [4]. It is well-known that the coordination of the metal ion plays a crucial role in the structure and stability of mononuclear enzymes, and the results herein suggested that metal B in dinuclear enzymes may play a similar structural role. Further research in more binuclear enzymes is needed to prove this proposal.

## 3. Experimental Section

### 3.1. Chemicals

Methylene blue, sodium dodecylsulfate (SDS), Tris, 1-anilinonaphtalene-8-sulfonate (ANS), isopropyl-1-thio-β-d-galactopyranoside (IPTG) and polyadenylic acid potassium salt with an average size of 200 adenosines were purchased from Sigma-Aldrich, Inc. Dithiothreitol (DTT) was BIOMOL product, and MOPS was from AMRESCO. Rnasin was purchased from Promega. Other chemicals were local products of analytical grade.

### 3.2. Site-Directed Mutagenesis

Site-directed mutagenesis was performed by using a QuickChange site-directed kit (Stratagene), and the primers were synthesized by Invitrogen. The primer oligonucleotides used were as follows: 5′-CGACTTCTTCGCCATCGCAGGGGAGTTTTCAGGAATC-3′ and its complement 5′-TTC CTGAAAACTCCCCTGCGATGGCGAAGAAGTCG-3′ for *parn*(D28A); 5′-CTTCGCCATCGA GGGGCGTTTTCAGGAATCAGTGATG-3′ and its complement 5′-CATCACTGATTCCTG AAAACGCCCCATCGATGGCGAAG-3′ for *parn*(E30A); 5′-GACACAATATGCTCTTGGCCGT CATGCACACAGTTC-3′ and its complement 5′-GAACTGTGTGCATGACGGCCAAGAGCATAT TGTGTC-3′ for *parn*(D292A); 5′-CCACGAGGCAGGCTACGCAGCCTACATCACAGGC-3′ and its complement 5′-GCCCTGTGATGTAGGCTGCGTAGCCTGCCTCGTGG-3′ for *parn*(D382A). The mutated genes were confirmed by DNA sequencing.

### 3.3. Protein Expression, Purification and Sample Preparation

The gene of the WT 54 kDa PARN was obtained as described elsewhere [20]. The recombinant WT and mutated proteins were expressed in *Escherichia coli* BL21(DE3)pLysS (Stratagene) and purified as described previously [12]. In brief, the overexpression of the recombinant proteins was induced by 0.1 mM IPTG at 16 °C. The proteins were first purified by Ni^2+^ affinity chromatography (Shenergy Biocolor BioScience and Technology), and then the final products were collected from an ÄKTA purifier equipped with a Superdex 200 HR 10/30 column (Amersham Pharmacia Biotech). The purity of the final products was above 98% estimated by SDS-polyacrylamide gel electrophoresis (SDS-PAGE) and size-exclusion chromatography (SEC) analysis. The protein concentration was determined according to the Bradford method using bovine serum albumin as a standard [37].

The proteins were dissolved in buffer T containing 20 mM Tris-HCl (pH 8.0), 100 mM KCl, 0.5 mM DTT, 0.2 mM EDTA and 20% (v/v) glycerol. The apoenzyme was prepared by chelating the binding Mg^2+^ by 2 mM EDTA, and then EDTA was removed by SEC purification and dialysis against buffer T overnight at 4 °C.

### 3.4. Activity Assay

The enzymatic activity was measured according to the standard methylene blue method as described previously [19] with some modifications [29]. In brief, the reaction buffer contained 20 mM Tris-HCl (pH 7.0), 1.5 mM MgCl_2_, 0.1 units of Rnasin, 0.5 mM DTT, 10% (v/v) glycerol and 0.1% BSA. After reaction in the dark at 37 °C for 30 min, the absorbance of the sample at 662 nm was measured on an Ultrospec 4300 pro UV/visible spectrophotometer at 30 °C. All activity data were the results of at least three repetitions.

### 3.5. Protein Thermal Aggregation

The aggregation of the samples was monitored by measuring the turbidity at 400 nm (A_400_) with an Ultraspec 4300 pro UV/Visible spectrophotometer using a 1 mL cuvette. The cuvette was sealed by PARAFILM during experiments. The final protein concentration was 0.2 or 0.1 mg/mL dissolved in buffer T. The experimental temperature was controlled by a water-bath. The aggregation kinetics was analyzed using Equation (1). Data fitting was performed by nonlinear regression analysis using the software Prism, GraphPad Inc. The errors of the fitted parameters were within 5%.

### 3.6. Spectroscopy

Far-UV circular dichroism (CD) spectra were recorded on a Jasco-715 spectrophotometer using a cell with a path length of 0.1 cm. Intrinsic fluorescence spectra were measured on a Hitachi F2500 spectrophotometer using a 0.2 mL cuvette with an excitation wavelength of 295 nm. The hydrophobic exposure of proteins was evaluated by adding a 50-fold molar excess of ANS to the protein solutions, and then ANS fluorescence was measured using an excitation wavelength of 380 nm after the samples had been incubated for 30 min in the dark. All spectroscopic experiments were carried out at 25 °C with a protein concentration of 0.2 mg/mL. The resultant spectra were obtained by the subtraction of the spectra of the buffer.

## 4. Conclusions

In conclusion, the effects of the four mutations on PARN thermal aggregation suggested that the four conserved acidic residues in the active site were important to PARN stability, while their distinct Mg^2+^ dependent properties implied that the two Mg^2+^ ions in the active site contributed differently to PARN structural integrity. The present study also suggested that alterations in the active site structure could result in a global conformational change of the molecule, which might be important to the understanding of the cooperation between the domains in PARN function.
